# Selective adaptation of German /r/: A role for perceptual saliency

**DOI:** 10.3758/s13414-022-02603-2

**Published:** 2022-12-07

**Authors:** Holger Mitterer, Eva Reinisch

**Affiliations:** 1 Department of Cognitive Science, Faculty of Media and Knowledge Sciences, University of Malta, Msida, MSD 2080 Malta; 2 Hanyang Institute for Phonetics and Cognitive Sciences of Language (HIPCS), Hanyang University, Seoul, South Korea; 3 Acoustics Research Institute, Austrian Academy of Sciences, Vienna, Austria

**Keywords:** Speech, Perception

## Abstract

In three experiments, we examined selective adaptation of German /r/ depending on the positional and allophonic overlap between adaptors and targets. A previous study had shown that selective adaptation effects with /r/ in Dutch require allophonic overlap between adaptor and target. We aimed at replicating this finding in German, which also has many allophones of /r/. German post-vocalic /r/ is often vocalized, and pre-vocalic /r/ can occur in at least three forms: uvular fricative [ʁ], uvular trill [ʀ] and alveolar trill [r]. We tested selective adaptation between these variants. The critical questions were whether an allophonic overlap is necessary for adaptation or whether phonemic overlap is sufficient to generate an adaptation effect. Surprisingly, our results show that both assertations are wrong: Adaptation does not require an allophonic overlap between adaptors and target and neither is phonemic overlap sufficient. Even more surprisingly, trilled adaptors led to *more* adaptation for a uvular-fricative target than uvular-fricative adaptors themselves. We suggest that the perceptual salience of the adaptors may be a hitherto underestimated influence on selective adaptation.

## Introduction

Selective adaptation refers to the phenomenon that repeated exposure to one type of stimulus leads to the perception of the “opposite” on a neutral stimulus. For instance, perceiving continuous downwards motion (as in a waterfall) leads to the illusion that stationary objects are moving upwards (Crane, 1988), seeing vertical lines in a red colour makes grey vertical lines appear greenish (McCollough, 1965), and, in speech perception, repeatedly hearing a /da/-like sound makes subsequent stimuli between /da/ and /ga/ sound more like /ga/ (Eimas & Corbit, 1973). The latter effect has seen a recent resurgence in interest, partly as it was re-interpreted as distributional learning (Kleinschmidt & Jaeger, 2015) and partly because of a resurgence of the debate about “units” in speech perception (Cutler et al., 2010).

While there is little doubt in visual-word recognition that letters represent an important pre-lexical unit (Coltheart et al., 2001; McClelland & Rumelhart, 1981), no such consensus exists in spoken-word recognition. Arguing for phonemes as a unit in spoken-word recognition, Bowers et al. (2016) found that adaptation to word-final stops (either /b/ or /d/) affects target stimuli – as we will call the stimuli on which the effects of an adaptor series are measured – with word-initial /b/ or /d/ (*dump* vs. *bump*). They explained this effect by assuming that both word-final and word-initial /b/ activate a /b/-phoneme detector, and more generally argued that spoken-word recognition involves position-invariant phoneme representations at a pre-lexical level.

One issue with that study was, however, related to the phonetic details of the stimuli. The adaptors with word-final stops (in British English) contained many released stops, leading to an acoustic overlap between the adaptors and target stimuli. Selective adaptation is generally understood to occur at multiple processing levels, including at auditory levels (Samuel & Kat, 1996), so that the adaptation between stops in onset and offset positions observed in Bowers et al. (2016) might be due to acoustic overlap of the stop releases in adaptor and target stimuli. This was tested by Samuel (2020), who found that the selective adaptation effect in these stimuli disappears when the stop releases of the word-final stops are edited out. In a similar vein, Mitterer et al. (2018) tested whether selective adaptation always occurs if there is a phonemic overlap between adaptors and target stimuli. They investigated two cases; in their first experiment, this was Dutch /r/. The target stimuli in that experiment made use of the approximant [ɹ] in offset position (the so-called Gooise ‘r’; see Van Bezooijen, 2005), while the adaptors either contained the same type of /r/ in the offset position or an alveolar trill in onset position. Selective adaptation was only found when the adaptor and the target stimuli contained the same allophone (i.e., the same variant of /r/), showing that phonemic overlap is apparently not sufficient to achieve selective adaptation. In their second and third experiment, Mitterer et al. (2018) made use of allophonic variation of the German palatal fricative /ç/, which is produced as a velar fricative [x] when preceded by a back vowel. Moreover, [ç] can occur as an allophone of word-final /g/ (Mitterer & Müsseler, 2013). The palatal fricative was used as a target stimulus and the experimental design fully crossed phonemic and allophonic overlap in the adaptors. A control condition was implemented with a set of words that contained neither the phoneme /ç/ nor a surface [ç]. A condition with phonemic and allophonic overlap contained adaptors with underlying /ç/ and surface [ç] (e.g., *Kranich*, English ‘crane’). A condition with phonemic but not allophonic overlap contained words with underlying /ç/ and surface [x] (e.g., [bux] *Buch*, English, ‘book’), and finally, a condition with allophonic but no phonemic overlap contained a list of words with underlying final /g/, which was pronounced as [ç]. This was possible since German words ending on /ɪg/ (e.g., /kønig/, *König,* English, ‘king’) can be produced with either final [ɪç] or final [ɪk]. Using /g/-final adaptors implemented as [ç] then provides adaptors that have the same allophone but a different underlying phoneme. Selective adaptation was similar for both adaptors containing surface [ç] and not moderated by phonemic identity. That is, the list of /g/-final adaptors produced with [ç] lead to as much adaptation as the list of words with phonemic overlap, that is, surface [ç] and underlying /ç/. In contrast, the list of adaptors containing the phoneme /ç/ but surface [x] failed to lead to adaptation and patterned with the control condition.

Given the importance of this finding and the subsequent discussion regarding the status of the phoneme in spoken-word recognition (Morais, 2021; Samuel, 2020), the present study set out to extend these findings with German /r/. German /r/, just as Dutch /r/, has various allophones with the most sonorant variant, a vocalized /r/ (usually transcribed as [ɐ]) only occurring post-vocalically in syllable coda position. In syllable onset position, /r/ can be produced as uvular fricative [ʁ], uvular trill [ʀ], or alveolar trill [r] (Wiese, 1996). The most frequent variant is the uvular fricative, and Llompart et al. (2021) showed that German listeners recognize /r/-initial words better when produced with a uvular fricative than with an alveolar trill.

In the present study, we used this variation of German /r/ to further evaluate the role of phonemic and allophonic overlap in selective adaptation. In three experiments the allophonic overlap between the adaptor and target stimuli was varied while keeping the phoneme the same (i.e., /r/; control was an adaptor list with unrelated phonemes). Specifically, we manipulated the phonetic (i.e., acoustic and articulatory) similarity between the allophones in adaptor and target stimuli. Table 1 provides an overview of the combinations of adaptors and targets. Based on the literature, two possible predictions were tested: If phonemes serve as a unit in spoken-word recognition (Bowers et al., 2016), selective adaptation should be found as long as there is a phonemic overlap between adaptor and target stimuli, hence in all our critical conditions relative to the control, even though this effect might be smaller in the allophonic-only match conditions compared to conditions with phonemic *and* allophonic overlap. If, however, allophones are the crucial units in spoken-word recognition (Mitterer et al., 2013, 2016, 2018; Mitterer & Reinisch, 2017; Reinisch et al., 2014), selective adaptation should only occur if adaptors and target stimuli share the same allophone. To preview our results, we support neither of these potential outcomes. Instead, our findings suggest that perceptual salience may be a hitherto underestimated influence on selective adaptation.

**Table 1:** Overview of the experimental conditions in the three experiments

Experiment #	Adaptor conditions ranked by overlap with test continuum (position)				Test continuum /r/ variant
1	Control	vocalized /r/ (postvocalic)	uvular fricative (onset)	alveolar trill (onset)	[rozə] – [lozə] alveolar trill
2	Control	alveolar trill (onset)	uvular trill (onset)	uvular fricative (onset)	[ʁozə] – [hozə] uvular fricative
3	Control	alveolar trill (word-medial)	uvular trill (word-medial)	uvular fricative (word-medial)	[ʁozə] – [hozə] uvular fricative

## General method

### Overview

Table 1 provides an overview of the adaptors and target-stimulus continua used in the three experiments. We roughly followed the procedure of Experiment 3 in Mitterer et al. (2018), which provided the clearest results in that study. This included a pre-test to determine the most ambiguous stimulus of the target continuum for each participant individually. Two additional stimuli were then chosen close to this individually most ambiguous stimulus so that participants heard a range of stimuli in their respective most ambiguous region that were still different enough to engage them in phonetic categorization. Small changes were made relative to the experiment of the previous study since the current experiments were run in a web-based format.

### Participants

Participants were recruited via the online platform *prolific.co* (approximately 30 participants per experiment, see below)*.* We restricted participants to an age range from 18 to 40 years and excluded participants who had indicated language-related disorders in their sign-up at *prolific.co* (i.e., this study did not request any medically related information from the participants). Participants were allowed to take part in only one of the three experiments.

### Stimuli

All stimuli were recorded by a female native speaker of German who can produce all three variants of onset /r/ that are common in German and were used in the present experiments (uvular fricative [ʁ], uvular trill [ʀ], and alveolar trill [r]). She naturally vocalized /r/ in post-vocalic position. We used two test continua, one from an alveolar trill in *Rose* [rozə] "rose" to a (light) /l/ in *Lose* [lozə] "lottery tickets" in Experiment 1 and one from a uvular fricative in *Rose* (now produced as [ʁozə]) to a glottal fricative in *Hose* [hozə] "pants" in Experiments 2 and 3. Test continua were generated using audio-morphing of natural utterances based on the time-aligned version of STRAIGHT algorithms (Kawahara et al., 1999). For both continua ([r] to [l] and [ʁ] to [h]), 21 morphs were generated by changing the morphing ratio from 0% to 100% /r/ in steps of 5%. Based on informal pretests, seven stimuli were selected for both continua that covered the most ambiguous region but also contained stimuli that were unambiguously perceived as /l/, /h/ and /r/ by native speakers of German.

For each of the different adaptor conditions, 26 words were selected that fitted the phonological criteria of each experiment and condition (see Table 1 for the conditions and Appendix Table 4 in the Appendix for the full list of adaptors). Adaptors with /r/ in initial or word-medial but syllable onset position were recorded with a uvular fricative [ʁ], a uvular trill [ʀ], and an alveolar trill [r]. Adaptors with /r/ in postvocalic syllable coda position were recorded with vocalized /r/. For the fricative set, tokens were selected that contained little amplitude variation and a clear stretch of frication. For the trills, tokens with clear amplitude modulation were selected. For the vocalized /r/ condition, we selected tokens with a clear formant movement towards the vocalized /r/.

Note that, in contrast to many other selective adaption paradigms, we only adapt /r/ and not the other end of the continuum (/l/ in first experiment, /h/ in the other experiments). Our control condition is a list of word without any of these phonemes. This provides a better comparison if a given set of adaptors leads to no adaptation. If the “control” list contains, for instance, /l/ in Experiment 1, selective adaptation would lead to an /r/-bias. Consequently, it would be difficult to say whether a difference between the control condition and an experimental condition is due to adaptation of /l/ by the control list or adaptation of /r/ by the experimental list containing some form of /r/. By using a control list of words without any tokens of /r/, /l/, or /h/, we avoid this problem.

### Procedure

The experiments were generated using the jsPsych package (de Leeuw, 2015) and relied mostly on the html-keyboard-response and the audio-keyboard-response plugins of jsPsych (the experiment files are available at the Open Science Framework site for this project: https://osf.io/2ebfv).

Each participant first performed a pretest in which each of the seven stimuli selected for the respective continuum was presented ten times. Response prompts always contained a picture and the full word, rather than just the letter names (i.e., <h>, <l>, and <r>), since responses to pictures and words may be a more natural reflection of normal speech perception (Krieger-Redwood et al., 2013; McMurray et al., 2008). The response prompts were presented for 500 ms before the onset of the target stimulus. Once participants chose one option via a keyboard press, the other option disappeared from the screen to provide feedback that the response has been registered.

After the pre-test an automatic regression analysis was performed on the empirical logit transform of proportions of /r/ responses^1^ with continuum step as predictor. The results of this regression were used to calculate the step closest to the midpoint to select the three stimuli from the continuum to be used as the test continuum in the main experiment. The two stimuli around the most ambiguous one were selected based on the regression weight for continuum step. The selection criterion was that participants would hear at least one logit unit difference (roughly 24% around a 50% midpoint) between the more /r/-like stimulus and the other phoneme of the continuum (i.e., /l/ or /h/ respectively) with the most ambiguous step falling in between.

After the pretest, participants performed the main task, which was a set of 40 blocks of hearing 25 adaptors, presented at a rate of one stimulus every 1.2 s, followed by two repetitions of the three target stimuli (i.e., the most ambiguous step plus the two stimuli slightly biased towards one of the response options). The presentation of test trials followed the same procedure as the pretest trials. To ensure attention to the adaptors, participants were required to perform a word monitoring task while listening. That is, before each adaptor block, they were shown a written word. During the adaptor block they were required to press the space bar if the word occurred in the stream of adaptors. This could happen not at all, once or twice. Before each adaptor block, a random list of the 25 adaptors minus the monitoring target was generated. If the target was to be present, it was randomly chosen to replace one adaptor.

All experiments used four different adaptor types (see Table 1) and each adaptor type was used in ten consecutive blocks. After each ten blocks of adaptor and target stimuli, participants had the chance to take a short break. The experimental session including the pretest and main experiment lasted approximately 45 min.

## Experiment 1

In this experiment, the target stimuli came from a continuum from an alveolar trill to an alveolar lateral (i.e., from *Rose* [rozə] "rose" to *Lose* [lozə] "lottery tickets"). The adaptor lists (see Table 1) were the control list, a list with alveolar trills, a list with uvular fricatives (both in word onset) and a list with vocalized /r/ (necessarily postvocalic in syllable coda).

### Method

#### Participants

We recruited 32 participants with ages ranging from 18 to 40 years (mean: 28 years); 20 were male and 12 were female. Their first language was German, and all participants grew up in Germany apart from one participant from Austria. The data from four participants were lost, since the data file was not saved in time before participants were redirected to *prolific.co* (despite an 8-s countdown after initializing the saving of the file to the server), leaving 28 data sets for analyses.

#### Stimuli, design, and procedure

The stimuli, design, and procedure followed the general methods.

### Results and discussion

**Fig. 1:**
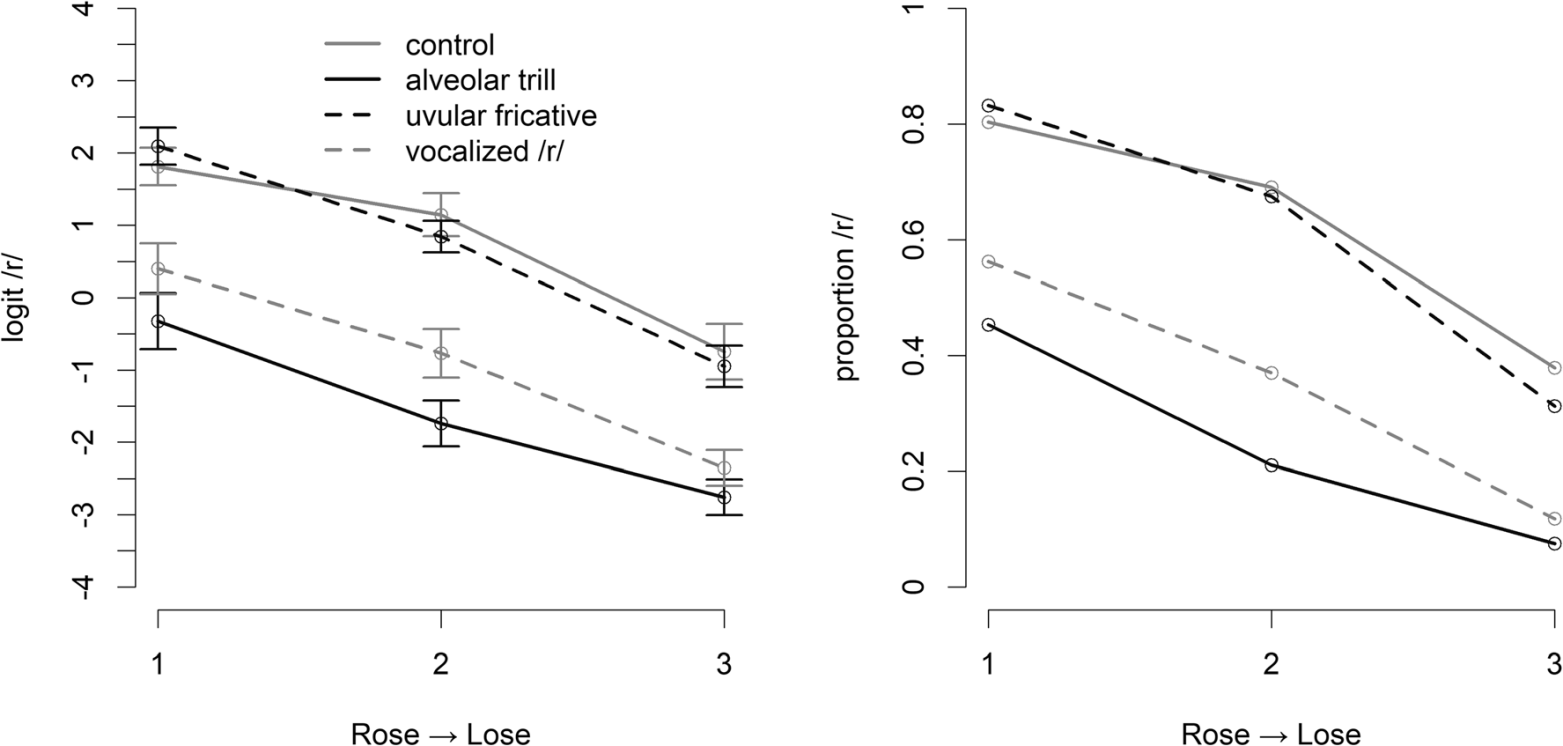
The proportion of /r/-responses (right panel) and their logit-transform (left panel) on the test trials in Experiment 1. The target stimuli were drawn from a continuum from /l/ to an alvoelar trill. Error-bars represent within-participant standard errors (Morey, 2008)

All participants reached more than 70% accuracy in the word monitoring task during the adaptor blocks, indicating that they paid attention to the adaptor stimuli. Figure 1 shows the proportion of /r/ responses in both logit units and proportions, with error bars indicating within-participant error bars (Morey, 2008) on the test continuum from an alveolar trill [r] in *Rose* to a an alveolar lateral [l] in *Lose*. The results shown in Fig. 1 indicate no difference between the control condition and a list of adaptors with a postvocalic, vocalized /r/. Both adaptors in the onset condition, however, appear to trigger selective adaptation. The effect for the alveolar trill adaptors that match the allophone of the target stimuli appears somewhat larger than the effect for the uvular fricative adaptors where the allophone does not match.

The results were analysed statistically using a repeated-measure ANOVA on the logit-transformed proportions of /r/ responses. This allows us to use the Bayesian approach of Dienes (2008) to transparently test whether a null result (no adaptation) is supported by the data. That is, we compare the null hypothesis against the hypothesis that adaptation with only phonemic but no allophonic overlap should be one third of the effect found in the full-overlap condition, as suggested by Bowers et al. (2016).

The ANOVA (using the aov_car function from the afex package, Singmann et al., 2021) with Adaptor (four levels; control, alveolar trill, uvular fricative, vocalized /r/) and continuum Step (three levels; i.e., the three steps of the test continuum from more /r/-like to more /l/-like) as factors reveals a main effect of Step (F(2,57) = 67.804, *p* < 0.001, p-values are based on a Greenhouse-Geisser correction for potential violation of sphericity), a main effect of Adaptor (F(3,81) = 37.269 , *p* < 0.001) and a significant interaction (F(6,162) = 2.789, *p* < 0.05). We therefore compared the differences between the adaptor conditions on each level of the continuum, using the lsmeans function (Lenth, 2018) with Holm correction for multiple comparisons. This showed that, firstly, there was no difference between the control condition and the vocalized-/r/ condition on any of the three levels of Step, secondly, that these two conditions differed from the two conditions with an onset /r/ (alveolar trill and uvular fricative) on all levels of Step (all *p*s < .001), and, thirdly, that the alveolar-trill condition differed from the uvular-fricative condition on only the first and middle level of Step (p < 0.05 and *p* < 0.01) but not on the final, most /l/-like step of the continuum.

To substantiate that there is no difference between the control condition (i.e., adaptors without /l/, or /r/) and the vocalized-/r/ condition, we generated a Bayes factor comparing the null-hypothesis with the hypothesis that the effect of phonemic overlap is one-third of the effect with phonemic and allophonic overlap. Following Dienes (2008), the experimental hypothesis was based on a normal distribution with one-third of the maximal effect generated by the alveolar trills as mean and a standard deviation of half that point estimate – so that values below zero are unlikely. A likelihood distribution for the null hypothesis was generated by a normal distribution with a mean of zero and a standard deviation based on the standard error of the mean differences between the control and the phonemic-overlap only condition. Comparing the likelihood of both hypotheses at the observed amount of selective adaptation in the control condition (-0.07 logit units), we get a Bayes factor of 0.07. This indicates that the null hypothesis is about fourteen times (i.e., 1/0.07) more likely than the alternative hypothesis, which is considered strong evidence in Bayesian statistics. That is, the data support the null hypothesis assuming no selective adaptation from vocalized /r/ adaptors on alveolar-trill target stimuli.

The results show a mixed picture with regard to our initial hypotheses. First of all, the results replicate the finding of Mitterer et al. (2018) that there is no selective adaptation between a strongly sonorant version of /r/ in coda position (an approximant in Dutch used in Mitterer et al., 2018, and the vocalized version used here) and a word-initial alveolar trill target. However, the results show clear selective adaptation without allophonic overlap for the uvular fricative in syllable onset, which was smaller than the full match alveolar trill adaptor condition on only two of the three continuum steps. One possible explanation for this finding is that the uvular variants of /r/ are more common than the alveolar trill and considered the standard in German (Llompart et al., 2021; Wiese, 1996). They may therefore have a privileged status in generating adaptation effects. To investigate this further, we tested whether alveolar trill adaptors can also lead to adaptation on a uvular-fricative target stimulus in Experiment 2. We also tested whether phonetic (i.e., articulatory and acoustic) similarity may play a role and used a condition with uvular-trill adaptors.

## Experiment 2

In this experiment, the target stimuli were drawn from a continuum from *Rose* (Engl., “rose”) produced with a uvular fricative (i.e., [ʁozə]) to Hose [hozə], with a glottal fricative /h/, which phonetically is the closest phoneme to the uvular fricative /r/ in German. As adaptors, we used (as previously) a control list of words not containing /r/, /l/, or /h/, a list of /r/-initial words produced with an alveolar trill [r], a list of /r/-initial words produced with a uvular trill [ʀ], and a list of /r/-initial words produced with a uvular fricative [ʁ].

### Method

#### Participants

We recruited 29 participants via *prolific.co*. Their age ranged from 20 to 37 years (mean: 26 years); 15 were male and 14 were female. Participants from Experiment 1 were not allowed to participate again in this experiment. Their first language was German, and all participants grew up in Germany apart from six participants from Austria. No data were lost in this study, since, after losing some data in the previous study, data were now also periodically saved to a secure mySQL data base during the experiment.

#### Stimuli, design, and procedure

The stimuli, design, and procedure followed the general methods.

#### Analysis

For the statistical analysis, we used a repeated-measure ANOVA on the logit-transformed proportions of /r/ responses to test effects of continuum step and adaptors. However, for the factor Adaptor we now employed three linearly independent contrasts (see Table 2) with the following rationale: A first contrast compared the control condition to all /r/ conditions, to see whether the experiment leads to selective adaptation overall (see row *Overall adaption* in Table 2). The second contrast (labelled *isTrill* in Table 2) compared the two trill conditions with the uvular fricative condition, based on the expectation that the uvular-fricative adaptors, which overlap with the target stimuli both phonemically and allophonically, lead to stronger effects than the two trill conditions, which overlap only phonemically. A final contrast investigated phonetic similarity by contrasting the alveolar-trill adaptor condition with the uvular trill adaptor condition (labelled *whichTrill* in Table 2), with the latter being phonetically more similar to the uvular-fricative target stimuli (i.e., sharing place of articulation). The contrasts were coded such as to provide the mean difference between the sets of conditions (see Table 2). Note that the conditions with less overlap are mapped on positive numbers, therefore, we expect positive regression weights from these contrasts because less overlap is expected to lead to *higher* proportions of /r/-responses (i.e., less adaptation).

**Table 2:** Contrasts (coding) for the adaptor condition in Experiment 2

	Adaptor condition			
	Control	Alveolar trill	Uvular trill	Uvular fricative
Overall adaptation	3/4	-1/4	-1/4	-1/4
Is trill	0	1/3	1/3	-2/3
Which trill	0	1/2	-1/2	0

### Results and discussion

**Fig. 2:**
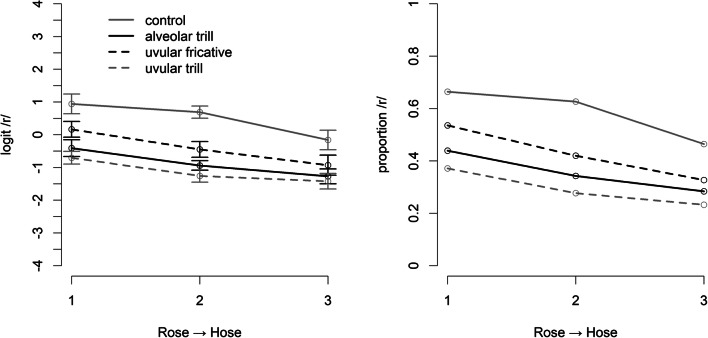
The proportion of /r/-responses (**right panel**) and their logit-transform (**left panel**) on the test trials in Experiment 2. The target stimuli contained a uvular fricative. Error-bars represent within-participant standard errors (Morey, 2008)

The data from one participant was removed because they scored less than 40% correct on the word monitoring task during the adaptation phase while all other participants scored above 90% correct responses. Figure 2 shows the descriptive data for the remaining 28 participants. It can be seen that all three versions of initial /r/ lead to selective adaptation, that is, they show a considerably smaller amount of /r/ responses than the control condition. Interestingly, even though the target stimuli contain a uvular fricative, the fully matching adaptors with the uvular fricative appear to produce somewhat less adaptation than the two conditions with trilled allophones (i.e., [ʀ] and [r]).

The statistical analysis revealed a main effect of continuum Step (F(2,54) = 10.699, *p* < .001), a main effect of Adaptor (F(3,81) = 20.346, *p* < .005), but no significant interaction (F(6,162) = 2.081, *p* = .085). The effect of the adaptor condition was further investigated using the contrasts discussed above. This revealed an overall adaptation effect of the three experimental conditions compared to the control condition of 0.96 logit units (SE = 0.152, t(27) = 6.378, *p* < .001), *less* adaptation (-0.40 logit units, SE = 0.141) with the uvular fricative adaptors compared to the trill adaptors (t(27) = -2.836, *p* < .01), and no significant difference between the two trill adaptors (t(27) = 1.489, *p* = 0.148).

These results are surprising, as they were predicted by none of the theoretical alternatives. Independently of any underlying assumption about phonemes in spoken-word recognition, the prediction was that the adaptors with the strongest overlap with the target stimuli – the uvular-fricative adaptors – should lead to the strongest adaptation effects. In contrast with this prediction, we find that trill adaptors (both alveolar and uvular) produce more adaptation on the uvular-fricative target stimuli than uvular-fricative adaptors themselves. As we discuss in more detail in the *General discussion*, trills are rather salient segments. It might be, with all adaptors in the onset, that the saliency of the trills may have repercussions at the decision stage. Remez (1987) had argued that adaptation may not only occur at levels of perception but also at the response level (see also Diehl, 1981), since adaptation can even be found for distinctions between speech and non-speech sounds (Remez, 1980). Since it is unlikely that there is a detector for non-speech, Remez (1987) argued that this adaptation must occur at the response level. This might be a possible explanation for the current finding. To further explore this possibility, we ran an additional experiment with the same adaptor conditions as in Experiment 2 but in which the adaptors occurred in word-medial position rather than in the (salient) word-initial position. This might reduce saliency-based effects at the response level. The stimuli from the test continuum were identical to Experiment 2 with the target sounds in word-initial position.

## Experiment 3

### Method

#### Participants

We recruited 29 participants via *prolific.co*. Their age ranged from 19 to 39 years (mean: 27 years); 14 were male and 15 were female. Participants from the earlier experiments were excluded from participating again in this study. Their first language was German, and all participants grew up in Germany apart from two participants from Austria.

#### Stimuli, design, and procedure

The stimuli, design, and procedure followed the general methods.

### Results

**Fig. 3:**
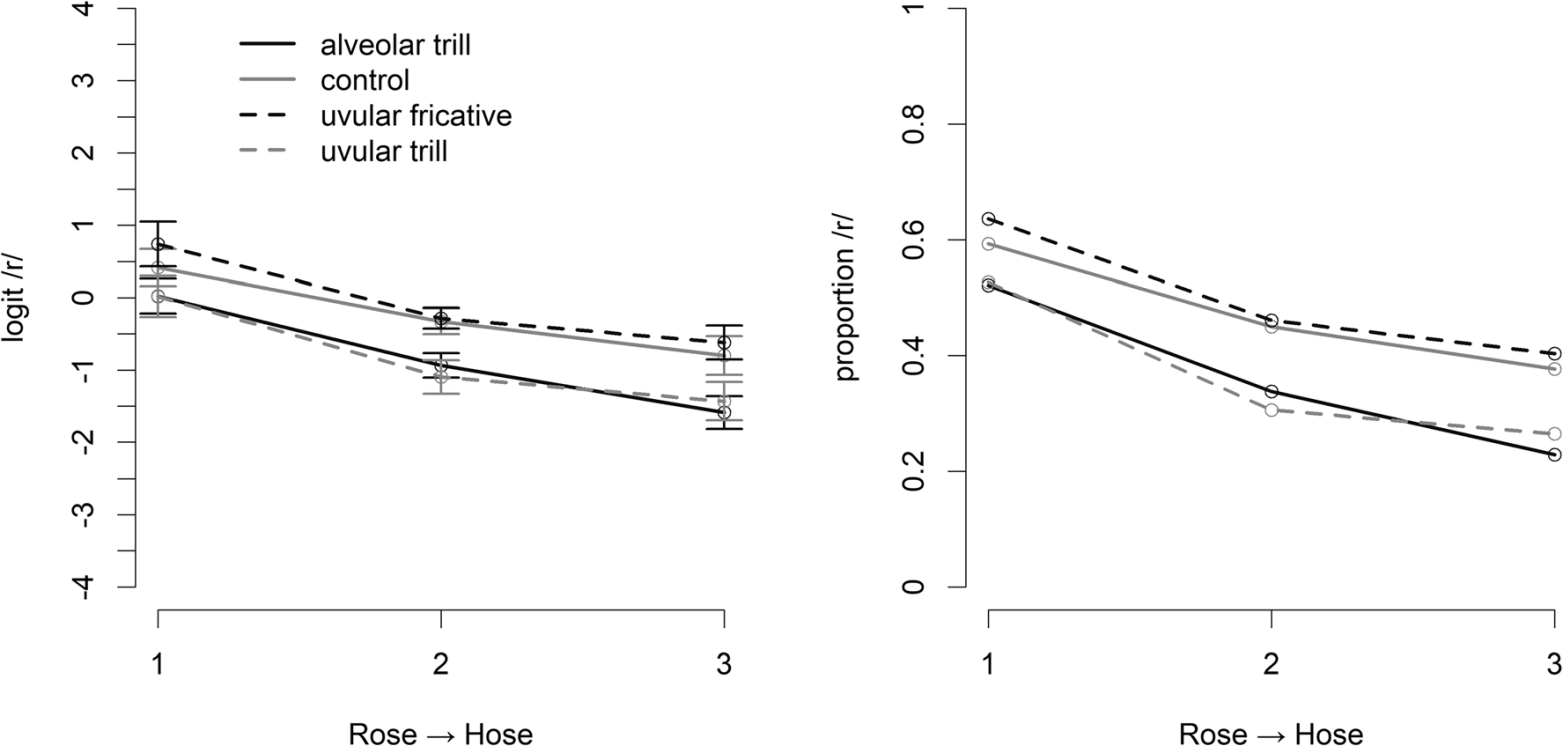
The proportion of /r/-responses (**right panel**) and their logit-transform (**left panel**) on the test trials in Experiment 3. The adaptors contained /r/ in word medial (syllable onset) position, the target stimuli were drawn from a continuum where the /r/ endpoint contained a uvular fricative in word-initial position. Error-bars represent within-participant standard errors (Morey, 2008)

In the word-monitoring task during adaptation, all participants spotted the target words correctly at least 70% of the time. Therefore, data of all 29 participants’ data were included in the analyses. Figure 3 shows the mean proportion of /r/ responses (right panel) and their logit transform (left panel) depending on continuum level and adaptor. Compared to the previous experiment, the figure suggests that selective adaptation is overall somewhat smaller when adaptor and target stimuli contain the /r/ in different word positions (though always in syllable onset). Moreover, the adaptors that phonetically match the test stimuli (i.e., uvular fricatives) here appear to fail in triggering selective adaptation. That is, they do not lead to fewer /r/ responses than adaptors of the control condition.

The statistical analysis was identical to the one described for Experiment 2 with contrasts for the levels of the factor Adaptor coded as shown in Table 2. It revealed a main effect of Step (F(2,54) = 17.502, *p* < .001), a main effect of Adaptor (F(3,81) = 9.794, *p* < .001), but no significant interaction (F(6,162) = 1.076, *p* > .2). Evaluating the contrasts for the adaptor condition revealed an overall adaptation effect of the three experimental conditions compared to the control condition of 0.25 logit units (SE = 0.093, t(27) = 2.668, *p* = .013), *less* adaptation (-.52 logit units, SE = 0.103) with the uvular fricative adaptors compared to the trill adaptors (t(27) = -5.090, *p* < .01), and no difference between the two trill adaptors (t(27) = 0.011, *p* > 0.2).

The finding that the adaptors with allophonic and phonemic overlap (the uvular-fricative adaptors) seem to pattern with the control condition is rather surprising. To investigate whether this is likely to be a robust null-finding, we again performed a Bayes-factor analysis, comparing the null-hypothesis with the hypothesis that the uvular-fricative produces as much adaptation than the alveolar trill.^2^ The resulting Bayes factor is 0.065, which is considered strong evidence for the null hypothesis.

In this experiment, we examined adaptor effects of syllable-initial but word-medial /r/ on /r/ in word-initial position. The results show a very similar pattern to those in Experiment 2, with more adaptation caused by trills than by a uvular fricative, even though the latter should, based on all theoretical considerations, be at least as strong an adaptor as a trill since it constitutes the full match condition to the target. Instead, the condition with the strongest phonetic overlap with the test continuum fails to show any sign of adaptation at all, despite solid adaptation effects in the two trill conditions.

## General discussion

We investigated selective adaptation of German /r/ to test to what extent adaptation is position specific and constrained by allophonic overlap. Table 3 provides a summary of the findings. Experiment 1 investigated selective adaptation on an alveolar-trill target in word-initial position by adaptors with word-initial alveolar trills, word-initial uvular fricatives, and post-vocalic vocalized /r/s, all compared to a control condition of words without /r/ (and without /l/, the other phoneme of the target contrast). Selective adaptation was observed only for the two word-initial /r/ allophones, with stronger adaptation for the alveolar trill that phonetically fully matched the target allophone. The proportion of /r/ responses in the word-final vocalized /r/ condition was similar to the control condition, indicating a lack of any selective adaptation as confirmed by a Bayes factor. Experiment 2 partly reversed the rolls of conditions, using alveolar trills as adaptors and a uvular fricative as target. Additionally, uvular fricatives and uvular trills – both in word-initial position – were used as adaptors to test a full allophonic match, and a phonetically closer allophone (matching place of articulation). Surprisingly, again the trills led to the strongest adaption, stronger than the adaptation by the fully matching uvular fricatives. This pattern was replicated in Experiment 3, in which the adaptors were in word-medial position with targets containing /r/ word initially (identical to Experiment 2). However, adaptation effects were generally smaller in Experiment 3 than in Experiment 2, suggesting that position overlap matters. Critically, and maybe surprisingly, in Experiment 3 the uvular-fricative adaptor condition patterned with the control condition.

**Table 3:** Overview of selective-adaptation effects in the current study

	/r/ target stimulus	Adaption effects
Exp1	[rozə] (alveolar trill)	[#rV…] > [#ʁV…] > […Vɐ(C)#] = ∅
Exp2	[ʁozə] (uvular fricative)	[#rV…] = [#ʀV…] > [#ʁV…] > ∅
Exp3	[ʁozə] (uvular fricative)	[#...rV…] = [#...ʀV…] > [#...ʁV…] = ∅

Note: “= ∅” means that an adaptor condition is similar to control according to a Bayes factor. “#” indicates a word boundary

These results reveal several patterns. First of all, as suggested by Mitterer et al. (2018) phonemic overlap between a list of adaptors and the target stimuli is not sufficient for selective adaptation to occur. We replicate this finding here twice. In Experiment 1, postvocalic vocalized /r/s failed to trigger selective adaptation for an alveolar trill as target allophone. In Experiment 3, only a difference in position (word-initial vs. word-medial uvular trills as adaptors and target stimuli) was sufficient to prevent selective adaptation. In both cases, a Bayesian analysis suggested that the null-hypothesis is more likely than the hypothesis that there is selective adaptation. However, in some cases, selective adaptation with only phonemic overlap was nevertheless observed. At first glance, this latter finding might be considered as evidence for phonemic representations. There are two reasons to doubt this conclusion, however. First of all, the assumption of phonemic representations in spoken-word recognition predicts that selective adaptation is *always* observed if there is a phonemic overlap between adaptors and target stimuli. With the current data and those by Mitterer et al. (2018) as well as Samuel (2020), there are now at least six demonstrations of a failure to find selective adaptation of phonemes across word positions. Samuel (2020) lists five other studies that mostly find non-significant or very small effects. Unfortunately, Samuel (2020) does not present a funnel plot of these earlier studies, so that it is difficult to say whether the abundance of non-significant effects is due to an underlying null effect or insufficient power of the individual studies. Most of those earlier studies are from the “first wave” of selective-adaptation studies (see Fig. 1 in Samuel, 2020) following the original report by Eimas and Corbitt (1973). In those studies, the sample size typically was between ten and fifteen participants, and sometimes as low as five (Ades, 1974).

In the more recent studies, however, the sample size was around 30 participants. This may seem small in comparison to the suggestion of sample sizes of forty advocated for by Brysbaert and Stevens (2018). However, Brysbaert and Stevens (2018) focus on reaction-time (RT) studies with samples of participants and items. In contrast, selective adaptation does not involve a sample of stimuli, and as a psychophysical method, typically has much lower variance than RT measures. Indeed, the Bayesian analyses showed that the data here lead to estimates of condition means that are sufficiently precise to provide evidence for the null hypothesis. As such, it seems that there is sufficient evidence that phonemic overlap does not consistently lead to selective adaptation.

Secondly, our results in Experiments 2 and 3 provide a pattern that is a challenge to explain for any theory. We find *more* adaptation from adaptors that overlap *less* with the target stimuli. That is, with uvular fricatives as target stimuli, alveolar and uvular trills consistently produced stronger selective adaptation effects than uvular fricatives themselves. This result is difficult to explain. If we assume that selective adaptation reflects overlap at the auditory, phonetic, and phonological level, it is difficult to see how trills can overlap on any of these levels more strongly with fricatives than fricatives themselves.

This leaves one level: the response level. Remez (1987) had already argued that selective adaptation may also occur at a response level. It is worthwhile to consider this avenue to explain our unexpected findings. It is possible to argue – based on well-accepted principles of perception – that trills are likely to capture attention more than other speech sounds due to their rapid amplitude modulation. Perception in general is perception of change (Kluender et al., 2003), which indicates that trilled speech sounds are likely to capture attention and hence be more salient. A similar conclusion is reached by Solé (2002, p. 682), who argues that trills are perceptually salient because their trilled manner of articulation results in "a clearly modulated signal, distinct from other speech segments”. If we additionally follow the assumption of Norris et al. (2000), that phonetic-categorization tasks can lead to the generation of meta-linguistic decision nodes for that task, we can start formulating how our results might be explained. It might be that such decision nodes are activated more strongly by salient trills than by less salient fricatives, which in turn might explain their strong effects on selective adaptation.

Invoking salience as an explanatory factor is not without issues, since salience can be a circular concept (MacLeod, 2015). Moreover, our account is entirely post hoc. Nevertheless, regarding change – as the primary characteristic of trills – leading to salience, there is a wide array of independent evidence to support this claim. Change is described as salient and driving perception in a wide array fields such as selective visual attention (Theeuwes, 1991; van der Heijden, 1992), visual illusions (Crane, 1988), and even measurements of neural activity in the auditory nerve (Delgutte & Kiang, 1984). Moreover, there are empirical conceptualizations of saliency based on both perceptual properties (such as sudden changes) and language properties (with rare features in a language being more salient) that allow one to narrow down the notion of salience (Auer et al., 1998; MacLeod, 2015). Alternatively, salience can be defined by independent observations, such as the likelihood of phonetic accommodation (Auer et al., 1998; Mitterer & Müsseler, 2013; Podlipskỳ & Simácková, 2015). To provide a new, independent test with another phoneme, it would be necessary to find a phoneme with two allophones that can be argued to differ in salience. This would be possible, for instance, in Dutch, where high, phonologically long vowels can surface as monophthong vowels or as slightly diphthongized (Mees & Collins, 1983). Given that diphthongized variants contain acoustic change, but the monophthong variants do not, it can be argued that the diphthong allophone is more salient than the monophthong allophone. Therefore, the prediction would be that the diphthongs lead to stronger selective adaptation than the monophthongs, even if the latter are the targets.

If indeed salience influences selective adaptation at the decision level, it would suggest that the use of the selective-adaptation paradigm to understand the structure of the spoken-word recognition system is less straightforward than currently assumed (e.g., Bowers et al., 2016). If post-perceptual levels strongly influence the results, which seems to be a viable explanation of our results, then great care is necessary in the interpretation of results from selective-adaptation studies. Such issues with the selective-adaptation paradigm are not new (Diehl, 1981; for a defence of the selective-adaptation paradigm, see Samuel, 1986). While the arguments for and against the strength of the paradigm indicate that selective adaptation cannot be reduced to decision-level effects, it does not rule out that decision-level effects contribute to selective adaptation. This would mean that for studying issues of pre-lexical units in speech perception, other experimental paradigms may be better suited. For instance, perceptual learning effects have been shown to target implicit processes of perception (McQueen et al., 2006; Sjerps & McQueen, 2010).

At this stage, one might wonder whether, by introducing selective adaptation at the decision level, a model of selective adaptation that assumes a phonemic level could explain our findings. While such a model with adaptation at the phonemic and at the decision level is clearly possible, it is still challenged by the current data. Such a model predicts some amount of selective adaptation at the phonemic stage in case of a phonemic overlap. The additional possibility of adaptation at the decision stage does not change the prediction that overlap at the phonemic stage should lead to some amount of selective adaptation. But as discussed above, several data sets, including the current one, suggest that phonemic overlap is not sufficient to cause selective adaptation, as models assuming such a stage – with or without additional decision stage effects – have to predict.

To summarize, we observe that patterns of selective adaptation clearly show that phonemic overlap is not sufficient to generate selective adaptation. As such, selective adaptation does not support the assumption of context-independent phonemic representations in word recognition. However, the data also show that phonemic overlap can sometimes lead to adaptation, despite lacking allophonic overlap. Given that this effect is sometimes stronger than effects with allophonic overlap, it seems that salience of the response categories in the input might be an important influence in selective adaptation.

### Open practices statement

The data, materials, and analysis scripts for all experiments are available at https://osf.io/2ebfv and none of the experiments were pre-registered.

## Appendix

**Table 4:** Adaptors used in the three experiments. Initial /r/ and medial /r/ were recorded with alveolar trills, uvular trills, and uvular fricatives, final /r/ items were only recorded with a vocalized /r/

Adaptor condition							
initial /r/		medial /r/		final /r/		control	
Rahm	cream	Barock	baroque	Bier	beer	acht	eight
Rand	edge	Beruf	profession	borgen	borrow	Affe	monkey
Raps	rapeseed	direkt	direct	Chor	choir	Bein	leg
rasch	fast	Geräusch	noise	Dekor	decor	Benzin	gasoline
Raste	notch	kariert	checkered	Erde	earth	Dampf	steam
Rauch	smoke	korrekt	correct	Firma	firm	Daumen	thumb
Rechen	rake	Morast	morass	Flur	hallway	Fest	fixed
Regen	rain	Pirat	pirate	Gurke	cucumber	Geige	violin
Reh	deer	Abriss	demolition	Kerbe	notch	Gift	poison
Reibe	grater	Anreiz	incentive	Kurve	curve	Kette	chain
reich	rich	Aufruf	call	mehr	more	Mai	May
Reis	rice	Nahrung	food	Ohr	ear	Monat	month
reisen	travel	Niere	kidney	Orbit	orbit	Nonne	nun
rennen	to race	Schere	scissors	Orkan	hurricane	Nuss	nut
Rest	remainder	Störung	fault	quer	across	Ost	east
Rezept	prescription	Virus	virus	Schirm	screen	Ozon	ozone
Riese	giant	Möhre	carrot	Schnur	cord	Post	Post
Ring	ring	Fischerei	fishing	Schwur	oath	Pumpe	pump
Robbe	seal	Apparat	apparatus	Speer	spear	Schiff	ship
Roggen	rye	Giraffe	giraffe	Teer	tar	Sonne	sun
Rom	Rome	Marine	marine	Tier	animal	Stock	stick
Rost	rust	aufräumen	clean up	Torte	cake	Tennis	tennis
Rübe	turnip	Dirigent	conductor	Uhr	clock	Topf	pot
Rum	rum	Parasit	parasite	vier	Four	Tusche	Indian Ink
Rummel	hype	Gitarre	guitar	werben	advertise	Wein	wine
rund	round	Kamera	camera	zirka	roughly	Zange	pliers

## Footnotes

1 In the study of Mitterer et al. (2018), a research assistant inspected the results of the pretest and then entered the choice when running the main experiments. This was not possible in the web-based format. Since the regression had to be performed (and programmed) in JavaScript, it was more tractable to perform a linear regression on transformed data than a logistic regression on the raw data.

2 Note that the prior of one-third of the maximal effect (which would lead to a BF of 0.321 in this case) is not a particularly sensible prior in this case. The “one-third prior” is based on the assumption that selective adaptation is based on allophonic and phonemic overlap (see Bowers et al., 2016) and hence a phonemic-only overlap effect should be considerably smaller than a phonemic-plus-allophonic overlap effect. Given that the uvular fricative condition has *more* overlap with the target stimuli (i.e., allophonic *and* phonemic) than the alveolar trill condition (phonemic overlap *only*), it is sensible to assume that the selective adaptation effect caused by uvular fricatives should be at least of similar size as the selective adaptation effect caused by alveolar trills.
